# Prader-Willi syndrome mental health research strategy workshop proceedings: the state of the science and future directions

**DOI:** 10.1186/s13023-016-0504-1

**Published:** 2016-09-29

**Authors:** Lauren Schwartz, Anthony Holland, Elisabeth Dykens, Theresa Strong, Elizabeth Roof, Jessica Bohonowych

**Affiliations:** 1 Department of Rehabilitation Medicine, University of Washington, 1959 N.E. Pacific St, Box 356490, Seattle, WA 98195 USA; 2 Department of Psychiatry, Cambridge Intellectual and Developmental Disabilities Research Group, University of Cambridge, Cambridge, UK; 3 Vanderbilt Kennedy Center, Vanderbilt University, Nashville, TN USA; 4 Foundation for Prader-Willi Research, Los Angeles, CA USA; 5 Department of Medicine, University of Alabama at Birmingham, Birmingham, AL USA

**Keywords:** Mental health research, Behavioral phenotype, Developmental disability, And intellectual disability

## Abstract

**Abstract:**

This paper reports on the ‘Prader-Willi Syndrome (PWS) Mental Health Research Strategy Workshop’ that took place in March 2015. PWS is characterized by a complex phenotype affecting multiple systems with a high prevalence of maladaptive behaviours, and neuropsychiatric illness. Prader Willi syndrome results from the absence of paternally derived alleles located at the imprinted chromosomal locus, 15q11–13. The goal of the workshop was to highlight the state of the science of the mental health of people with this rare neurodevelopmental disorder. Mental ill health and maladaptive behaviors significantly impact quality of life for persons with PWS and their caregivers. Effective treatments and further research into this area are critically needed.

**Methods:**

A multidisciplinary group of scientists and health care professionals were brought together to discuss the mental health and behavioral needs of people with PWS. The workshop strategy was to integrate established work on PWS with other relevant areas of study. The meeting also focused on two neurobiological systems that research had suggested were relevant to understanding the broader mental health aspects of PWS: the autonomic nervous system and oxytocin/vasopressin pathways. Other relevant topics were considered and recommendations made.

**Results:**

The workshop presentations and working group discussions revealed that no one approach was sufficient to fully conceptualize the mental health challenges in PWS. Workshop discussions pointed to the need for theoretically informed studies focused on clinical characterization, measurement, and the probing of specific neurobiological systems through pharmaceutical or other interventions. Future studies in this area should explore the use of advanced neuroimaging protocols, as well as molecular studies using iPS cells in order to create more informed theories.

**Conclusions:**

Within this framework, workshop participants identified and prioritized key research questions, and highlighted current opportunities. Recommendations were made with respect to the development of specific resources and tools for furthering mental health research such as The Global PWS Registry, the development of effective endpoints, the use of animal models and iPS cells to aid understanding of the neurobiological underpinnings. Additionally, collaborative opportunities across disciplines and syndromes were highlighted and targeted research initiatives focused on psychological/behavioral interventions modified for use in PWS were recommended.

## Background

PWS is a rare, genetically determined neurodevelopmental disorder characterized by a complex phenotype affecting multiple systems with a high prevalence of maladaptive behaviors, including neuropsychiatric illness. As is the case with many rare neurodevelopmental syndromes of genetic origin, PWS is associated with specific physical features and a characteristic developmental profile that make up the core diagnostic criteria for the disorder. There are also high rates of specific problem behaviours and psychopathology, which have become known as the ‘behavioural phenotype’ of the syndrome [1–3]. The aims of previous research have been to characterize these problems and to then identify the developmental, biological, and psychological mechanisms that directly or indirectly link the genotype to this phenotype. The central aim of the ‘Prader-Willi Syndrome (PWS) Mental Health Research Strategy Workshop’, which was held in 2015, in Bethesda, Maryland, was to review the state of the science and develop a strategy for moving the field forward. The workshop included clinicians and scientists active in mental health research into PWS and/or other neurodevelopmental syndromes, as well as family members, some of whom have also been actively engaged in research. In this paper we report on the proceedings of the workshop, providing an authoritative overview that identifies key target areas in need of comprehensive study and considers potential methodologies for pursuing such studies. The following sections will provide highlights from the workshop and will summarize the formal presentations, interactive sessions and final discussion session outlining workshop recommendations.

### Overview of the workshop

At the opening reception Lauren Schwartz, PhD (University of Washington) discussed the behavioural complexities of people with PWS, and the challenges in helping them navigate through life. The urgency and importance of mental health challenges in PWS was emphasized from a parent and clinician perspective. Elisabeth Dykens, PhD (Vanderbilt University) highlighted the breadth of PWS symptoms arguing that this both required, and should encourage, more psychiatric investigators to study PWS. She emphasized not only the importance for people with PWS and their families that we better understand these problems but also the value of PWS as an ideal, but under-recognized, model for elucidating the biological underpinnings of psychopathology in the general population. A “Work in progress” poster session followed these presentations. The posters focused on a range of relevant topic areas including: further characterization of the behavioural and psychiatric phenotype; learning strengths and challenges in PWS; behavioral interventions for anxiety, obsessive compulsive disorders, and aggressive behaviors in PWS; unique responses seen in individuals with PWS to psychiatric medications; neuroimaging studies; and the use of induced pluripotent stem cells to investigate the molecular basis of psychopathology in PWS.

The first full day of the workshop began with an overview of mental health in PWS followed by presentations from experts in related fields of mental health and developmental disability research. The goal of these talks was to identify how to apply knowledge in related areas towards new ways of conceptualizing and addressing mental health in PWS. The Workshop also included two highly interactive sessions addressing provocative questions in PWS mental health: “Can We Prevent or Mitigate Psychosis in PWS?” and what was referred to as “The Oxytocin Conundrum”. Finally, afternoon breakout sessions focused on the identification of key issues and research priorities within five topic areas, with a final summary and discussion session for consensus recommendations.

### Current state of the science

‘The problem behaviours and psychopathology in PWS’ was presented by Anthony Holland, M.D., (University of Cambridge). Dr. Holland outlined the current understanding of the features of PWS over the lifespan highlighting changing areas of need with respect to the eating behavior/hyperphagia, other problem behaviors and the propensity to mental illness. PWS is a genetically determined neurodevelopmental disorder associated with a phenotype that strikingly changes early in the lifespan [2]). Briefly, infants with PWS invariably fail to thrive and are hypotonic at birth and unable to suckle. Children are usually slow to develop with evidence later in childhood of an intellectual impairment and learning disabilities. Over-eating/hyperphagia also begins to develop in young children with five relatively distinct phases now described [4]. The hyperphagia is considered to be due to an impaired satiety response and a high reward value of food for individuals with PWS [5–7]. As children with PWS develop, characteristic physical features of PWS become apparent but these are largely minimized by growth and sex hormone replacement [8]. Adolescents and adults are particularly at risk for severe obesity because of the propensity for over-eating and increased opportunities to obtain food in different work or community settings. The hyperphagia and the relative growth and sex hormone deficiencies of pituitary origin, together with irregularities of sleep, temperature regulation and the report of reduced numbers of oxytocin containing cells in the para-ventricular nucleus, highlight the central role of the hypothalamus in the core aspects of the PWS phenotype [9].

The life course of people with PWS is also associated with a high risk for specific maladaptive behaviours and neuropsychiatric disorders, which are not unique to PWS, but are more prevalent than in other neurodevelopmental disorders associated with intellectual disabilities. Rates of psychopathology are high and generally remain so over time [10]. This PWS ‘behavioural phenotype’ includes: a) repetitive and ritualistic behaviours [11] similar to those found in autistic spectrum conditions [12]; b) temper outbursts that tend to occur at times of unexpected or unwanted change [13]; c) skin picking or other forms of self-injury that vary in nature and intensity [14]; d) mood disorders that can be cyclical in nature and characterized by both hypomanic and depressive phases [15]; and e) psychotic illness, mainly in people with PWS due to maternal uniparental disomy (mUPD) of chromosome 15, which is predominately affective in nature and may develop as early as the teenage years with the occurrence of abnormal mental beliefs and experiences [15–17].

These behavioural and psychiatric problems are found across all three of the major PWS genotypes (Type I or II deletions at 15q11–13, mUPD) with some differences in intensity or degree of problems across the lifespan [18, 19]. The most striking difference with respect to the different genotypes is the very high risk for affective psychosis seen in adults with PWS mUPD, with approximately 60 % displaying psychosis that usually presents in the late teens or early adulthood [16, 17, 20]. In contrast, psychotic illness is less often found in those with PWS due to deletion (~20 %), with depression reported in both genetic subtypes (20–25 %, [20]).

It has been proposed that the increased liability to non-psychotic mood disorders in PWS may be due to the absence of the snoRNA, SNORD115 (previously called HBII-52) and its effect on the serotonin 2C receptor [21, 22]. However, psychotic illness, which is more common in those with mUPD, may be a consequence of a second ‘genetic hit’ due to the excess expression of paternally imprinted gene(s) located on chromosome 15 [15]. In one of the few follow up studies on psychotic illness in PWS [23], contacted 48 individuals who had taken part in a previous study and had had episodes of psychosis at that time or were at increased risk of developing psychosis due to their genetic subtype. The [23] study found among the 28 adults who agreed to participate in the follow up, that recurrent episodes of psychosis were relatively uncommon (2 out of 28 participants had recurrent illness) once stability had been achieved with psychiatric medication. The medications included antipsychotic medication, SSRIs and/or mood stabilizers, used singularly or in combination. While standard psychiatric treatments were used to treat the psychotic illness with reasonable effect, the question remains whether from a pathophysiological perspective, the affective and psychotic illness in PWS should be seen as similar to or different from similar psychiatric illnesses in the general population.

The two main genetic subtypes of PWS also differ in intellectual profiles and level of social functioning. The deletion group generally having better visuo-spatial skills, while the mUPD group has higher verbal functioning [24, 25]. Those people with PWS due to mUPD have more features of autism spectrum disorders [26] and have also been shown to be slower at cognitive processing [27]. The molecular mechanism(s) underlying these differences in phenotype remains to be determined. In clinical practice the implications of such observations for direct support staff and clinicians is the recognition of the potential benefit of specific support strategies and, where appropriate, ‘autism informed’ interventions to minimize problem behaviours.

While the PWS behavioural phenotype is now well recognized it is not yet clear whether there are shared or separate mechanisms that directly or indirectly link the PWS genotype to different aspects of the behavioural phenotype. Factor analysis suggests three groupings of behaviours: a) eating disorder, lying and stealing; b) repetitive and ritualistic behaviours and temper outbursts; and c) skin picking and mood disorders. Each of these three groups may have different causal mechanisms [28]. For example, impaired set-shifting ability and different patterns of brain activation have been shown to be associated with increased temper outbursts, when routines are disrupted, and with repetitive and ritualistic behaviours [13, 29, 30]. Interestingly, the notion that environmental predictability is an important part of the management of such behaviours has been challenged, proposing instead that such adherence to routine may lead to more severe outbursts when those routines are then subsequently broken [31].

The key message of these examples of more recent research is that identifying possible neural and psychological mechanisms linking genotype to the PWS behavioural phenotype allows for more targeted and effective treatments. While structured and behavioural observations over time are critical, understanding the basis for such observations is clearly enhanced by combining with advanced neuroimaging. Studies to elucidate the behavioural and mental health needs of people with PWS can also take other forms including the use of specific interventions aimed at ‘probing’ particular neurotransmitter systems. An example of this can be seen in the open label study of N-acetylcysteine, a modulator of the excitatory glutaminergic pathway, in which Miller and Angulo [32]) reported reductions in the extent and severity of skin picking. Other emerging areas of therapeutic investigation that are redirecting attention to different explanations for such behaviours include oxytocin for social impairments and anxiety (see below), vagus nerve stimulation for behavioural difficulties [33], behavioural interventions including mindfulness and cognitive behavior therapy, and detailed functional analyses such as been undertaken with skin picking [14].

From a clinical perspective the state-of-the-art remains that of a detailed assessment that brings together perspectives of applied behavioural analysis and the identification of physical and psychiatric co-morbidities. This assessment also must focus on identifying the interplay of developmental behaviours, acquired co-morbid illness, and/or other disturbances (e.g. sleep disorders) with factors in the environment that might predispose, precipitate and maintain the problematic behaviours or abnormal mental state (see best practice guidance, [34]).

### Lessons from other neurodevelopmental disorders and specific neurobiological systems

Tony Simon, PhD (University of California, Davis), presented “Cognitive-affective interactions and psychosis risk (and Protection?): The case of 22q11.2 deletion syndrome”. This particular syndrome was chosen as PWS and 22q deletion share many features, including developmental delay, increased anxiety, and high risk of psychotic illness. Dr. Simon explained that individuals with 22q deletion can be separated into two groups based on their level of anxiety– those who manage anxiety well, termed “copers”, and those battling their anxiety, termed “strugglers”. This distinction between coper and struggler is independent of IQ, and instead correlates with allostatic load, or repeated and chronic stress [35]. Copers do better than strugglers in real world functioning and may outperform expectations based on cognitive testing alone, while the opposite is true for strugglers. “Strugglers” appear to be at greater risk for mental illness as allostatic load is also correlated with psychiatric difficulties in many populations ([35, 36]. Given these findings the importance of creating environments to help children become “copers” by speaking to children’s strengths and decreasing allostatic load/stress levels was clearly apparent. One approach is matching the person’s abilities to the demands of a particular situation or task. This may be accomplished with a variety of tools and approaches –employing behavioral therapy, creating an appropriate and supportive educational environment, using medication as needed, and setting realistic expectations for families and schools. A key question is whether the anxiety/coper/struggler paradigm also applies to our understanding of people with PWS.

Stephen Porges, PhD (University of North Carolina) spoke on “The polyvagal theory and autonomic nervous system function: implications for the understanding of the behavioural phenotype of PWS”. This topic had been selected as results from a small study on the use of vagus nerve stimulation in people with PWS had found significant improvements in behavior (Manning [33]). The polyvagal theory [37] is focused on the flight, fight or freeze responses to a real or imagined threat, and the impacts on social behavior and psychological functioning. Previous research drawing on the polyvagal theory has provided insights into the mechanisms mediating symptoms observed in several behavioral, psychiatric, and physical disorders as well as in autism [37–39]. Porges proposes that defects in the vagus system specifically in the “vagal break” may be related to deficits in social communication seen in PWS. These proposed defects may also underlie or trigger anxiety and temper outbursts. Future research is needed to determine how this model may highlight mechanisms in PWS that are disrupted and point towards novel treatment approaches for mental health challenges. The results from the Manning et al. [33]) pilot study indicate that the thresholds for when these behaviours are triggered by environmental events may possibly be moderated through manipulation of the vagus nerve as part of the autonomic nervous system. Further research into this pathway in PWS is warranted.

The final keynote presentation focused on the importance of measurement in clinical trials drawing upon work in Fragile-X syndrome (FXS) conducted by Elizabeth Berry-Kravis, MD, PhD (Rush University Medical Center) and colleagues [40, 41]. Dr. Berry-Kravis presented “Outcome measures for treatment trials in fragile X syndrome: hurdles, lessons and progress”. During this presentation, Dr. Berry-Kravis reviewed information from her previously published paper [40] on measuring endpoints and outcomes in clinical trials for Fragile X. Dr. Berry-Kravis detailed what has and has not worked for the FXS clinical studies [40, 41] and outlined ideas regarding ways the PWS research community could successfully adapt and develop effective measures to be used in PWS treatment studies. Several concepts were emphasized including taking the time to develop appropriate endpoint measures and the need for intense collaboration among researchers, clinical teams and caregivers. Global registries were identified as excellent resources to collect data to inform such endpoint development particularly for rare diseases.

### Interactive sessions

The first interactive session entitled “*Can we prevent or mitigate psychosis in PWS*?” focused on whether a clinical trial is warranted targeted at preventing the onset or recurrence of psychosis in PWS. The session was moderated by Anthony Holland, MD who summarized what is known about the onset of psychosis in PWS. Questions were then put to the group, is a clinical trial warranted? If so, what should the intervention be? What ethical issues need to be addressed? How would such a trial be conducted? After a thoughtful discussion, a general consensus was reached that better identification and characterization of persons with PWS who are at most risk is needed prior to a trial. There was strong consensus that current efforts should focus on better identifying those subgroups and on characterizing the “prodromal” symptoms of psychiatric illness in PWS. Finally, determining the underlying biological mechanism of psychosis would help in designing an optimal clinical trial. Various ways of approaching such studies were discussed as well as various intervention modalities including psychiatric medication, omega-fatty acids [42] and cognitive therapy [43].

“*The Oxytocin Conundrum*” Long time expert in the area of oxytocin research, Sue Carter, PhD (Indiana University) presented an overview of the research on the biology of oxytocin. The usefulness of intra-nasal oxytocin in PWS remains uncertain as findings from initial clinical trials exploring the use of oxytocin in PWS are mixed [44], found positive effects of intra-nasal oxytocin on social functioning and behavior in PWS. However, no differences were found in a double blind placebo controlled trial [45], with worsening of temper outbursts found at the highest dose of oxytocin. Despite these seemingly conflicting results, there remained interest among the PWS researchers to continue to explore the potential utility of this medication for PWS. Group discussion focused on possible new ways of understanding how the oxytocin and vasopressin systems may be disrupted in PWS. Workshop participants identified key issues to address in future research including examining deficits in long acting and intermediate forms of oxytocin and the resultant effects on vasopressin receptors, as well as identifying critical windows for oxytocin action in the developing brain. Additionally, dosing issues were recognized as likely to be crucial for a positive effect and the timing of that dosing will likely vary across age groups of persons with PWS. Finally, Dr. Carter emphasized that in general lower doses of oxytocin at critical windows may be more beneficial than higher doses [46].

### Identification of Key issues

During the second part of the meeting, Workshop participants were each assigned to moderated working groups. The working group discussion topics focused on a series of pressing mental health challenges in PWS. These mental health areas of most pressing need were identified before the workshop via a survey completed by researchers and parents/caregivers. The top five topics identified via this survey were: 1) depression/mood issues, 2) Anxiety/Obsessive Compulsive behaviours, 3) Temper outbursts, 4) Social challenges and 5) further developing the Research Infrastructure for PWS mental health research. The moderators of each of the groups synthesized the discussion into a brief summary for final presentation and discussion with the full group. Table 1 summarizes the top 10 consensus recommendations from the working groups. These can be divided broadly into three areas: 1) the development of the necessary resources and research tools/strategies to enable the research – this was as broad as the use of the Global PWS registry to better understand the natural history of mental health and behavior in PWS, the development of outcome measures to support clinical studies and trials, and the use of animal models or iPS cells derived from people with PWS to better understand the neurobiological underpinnings of these disorders; 2) the advancement of theories and the research strategies directed towards testing such theories, with a particular focus on the oxytocin/vasopressin and autonomic nervous systems; and 3) the undertaking of clinical trials of psychological and other interventions both to inform clinical practice and also as a means of better understanding etiology.

**Table 1:** Top 10 research priorities from the PWS Mental Health Workshop

1. Obtain longitudinal and natural history data on mental health in PWS, including the behavioral and psychiatric components of the PWS phenotype. The Global PWS Registry will be a critical resource to advance mental health research since it will compile data on mental health over the lifespan.
2. Develop effective outcome measures for PWS mental health treatment studies, fully utilizing the Global PWS Registry to facilitate the development of such tools. 3. Determine the influence of weight management, hormones, and environment on mental well being over the life course, using validated measures of well-being. 4. Apply diverse research methods to advance mechanistic research on the neurobiology underlying mental health and behavioral issues in PWS. 5. Identify markers (neurobiological, genetic, psychological, environmental) of impending mental illness and characterize features of the prodromal phase (especially psychosis) in PWS, to allow mitigation and/or prevention of psychiatric episodes through environmental, behavioral or pharmacological interventions; and evaluate effectiveness of such interventions. 6. Facilitate further research on oxytocin and vasopressin in PWS in order to refine the timing and dosing of future oxytocin trials in PWS. 7. Further assess autonomic nervous system function in PWS to determine if the polyvagal theory and treatments help explain or ameliorate temper outbursts, anxiety and other behavioral problems. 8. Adapt or modify current state of the art behavioral interventions, including mindfulness, for anxiety, obsessive compulsive behaviors, and temper outbursts for PWS, and test their effectiveness over time. 9. Develop accessible methods and approaches to help reduce high levels of parental/caregiver stress, and determine effects of reducing caregiver distress/stress on child functioning. 10. Involve parents, families and caregivers (both family and professional) at all levels; Workshop attendees felt that their vast experience and engagement will be critical to advancing knowledge and treatment, with input as well from youth and adults with PWS.

## Conclusion

Individuals with PWS represent a genetically defined population with a high incidence of maladaptive behaviours and increased susceptibility to psychiatric illness. Understanding the basis of the phenotype in this rare disorder is likely to provide insight into more common forms of mental health concerns in the general population. It is hoped that this PWS Mental Health Research Workshop Summary will give researchers and clinicians a framework for understanding some of the key mental health challenges in PWS and the state of current research. Further, workshop recommendations can guide funding agencies in prioritizing future mental health research initiatives, thereby streamlining resources. Strengthening collaboration among the outstanding investigators at the meeting as well as additional experts in the field and advocates will be critical to advancing this research agenda.

## Abbreviation

PWS: Prader Willi syndrome
